# Determining the Acceptance of Digital Cardiac Rehabilitation and Its Influencing Factors among Patients Affected by Cardiac Diseases

**DOI:** 10.3390/jcdd10040174

**Published:** 2023-04-17

**Authors:** Alexander Bäuerle, Charlotta Mallien, Tienush Rassaf, Lisa Jahre, Christos Rammos, Eva-Maria Skoda, Martin Teufel, Julia Lortz

**Affiliations:** 1 Clinic for Psychosomatic Medicine and Psychotherapy, University of Duisburg-Essen, LVR-University Hospital Essen, 45147 Essen, Germany; 2 Centre for Translational Neuro- and Behavioral Sciences (C-TNBS), University of Duisburg-Essen, 45147 Essen, Germany; 3 Department of Cardiology and Vascular Medicine, West-German Heart and Vascular Center Essen, University of Duisburg-Essen, 45147 Essen, Germany

**Keywords:** UTAUT, mHealth, cardiac disease, internet, rehabilitation

## Abstract

Background: Cardiac diseases are a major global health issue with an increasing prevalence of affected people. Rehabilitation following cardiac events is underutilized, despite its proven effectiveness. Digital interventions might present a useful addition to traditional cardiac rehabilitation. Aims: This study aims to assess the acceptance of mobile health (mHealth) cardiac rehabilitation and to investigate the underlying factors of acceptance in patients with ischemic heart disease and congestive heart failure. Methods: A cross-sectional study was conducted from November 2021 to September 2022 with *N* = 290 patients. Sociodemographic, medical, and eHealth-related data were assessed. The Unified Theory of Acceptance and Use of Technology (UTAUT) was applied. Group differences in acceptance were examined and a multiple hierarchical regression analysis was conducted. Results: The overall acceptance of mHealth cardiac rehabilitation was high (*M* = 4.05, *SD* = 0.93). Individuals with mental illness reported significantly higher acceptance (*t*(288) = 3.15, *p_adj_* = 0.007, *d* = 0.43). Depressive symptoms (β = 0.34, *p* < 0.001); digital confidence (β = 0.19, *p* = 0.003); and the UTAUT predictors of performance expectancy (β = 0.34, *p* < 0.001), effort expectancy (β = 0.34, *p* < 0.001), and social influence (β = 0.26, *p* < 0.001) significantly predicted acceptance. The extended UTAUT model explained 69.5% of the variance in acceptance. Conclusions: As acceptance is associated with the actual use of mHealth, the high level of acceptance found in this study is a promising basis for the future implementation of innovative mHealth offers in cardiac rehabilitation.

## 1. Introduction

Cardiovascular diseases (CVDs) are a global health issue. In 2019, about 17.9 million people died from CVDs, making CVDs the leading cause of death worldwide [1]. Ischemic heart disease (IHD) and congestive heart failure (CHF) are the most common CVDs [2] with a prevalence of 197.2 million [3] and 64.3 million, respectively, worldwide [4]. Reasons for the ongoing rise in prevalence are ageing [5], as well as the increase in controllable CVD-risk factors, such as obesity [6] and diabetes [7]. Furthermore, mental health disorders are often associated with CVDs [8].

Cardiac rehabilitation (CR) supports patients with cardiac diseases (CD) by improving relevant outcomes, such as re-hospitalization rates, morbidity and mortality, quality of life (QoL), and associated healthcare costs [9,10]. CR is often characterized by a multi-modular concept, including an increase in physical activity and a general behavioral change towards a healthier lifestyle. The inclusion of stress reduction strategies is gaining more and more importance as a component of CR [11].

Despite its proven efficacy, CR is underutilized by patients, although there is a recurrence rate of nearly 50% for each cardiovascular event in the first year after an acute cardiac event [12]. Only about 13.9% of patients participate in CR after acute myocardial infarction [13].

Telerehabilitation might represent a good opportunity to increase the uptake of CR, through its easier accessibility. It includes digital offerings of established CR interventions, which are delivered via smartphone (mHealth interventions), web-based platforms, or videoconferencing systems. Telerehabilitation has the potential to support patients to establish long-term lifestyle changes and to help patients adhere to recommendations through increased self-monitoring [14,15].

Telerehabilitation is a cost-effective alternative to conventional CR and can serve as an adjunct to conventional CR or potentially as a replacement [16,17].

Adherence to treatment plays a role, especially in the secondary prevention of CVDs. Improving it is a complex problem and requires a multidisciplinary approach, which every health professional should adhere to. Many factors related to the patient, the disease, the healthcare provider, the therapy, and the healthcare system play a role in adherence [18]. Patients who tend to be non-adherent need to be identified, a multidisciplinary intervention pathway should be developed and an appropriate follow-up strategy should be established [18]. For this purpose, telerehabilitation could be an asset. It has higher uptake, adherence, and completion rates than traditional CR [19]. Physiological outcomes (e.g., walking distance) and psychological outcomes (e.g., depression and anxiety symptoms) were improved. CR effectively provides support to implement healthy lifestyle changes and to improve QoL. Hospital readmissions were reduced after CR [20]. Another meta-analysis demonstrated that the additional use of mobile applications can increase adherence in center-based CR, as well as minimize health problems [21].

Patients’ acceptance is essential when introducing new treatment approaches. To date, no study has assessed the acceptance of mHealth CR and its predictors with validated measurement methods in patients with CD. For this reason, the Unified Theory of Acceptance and Use of Technology (UTAUT) is used in the present study [22]. The UTAUT assesses the acceptance of technological systems and has been adapted to examine the acceptance of eHealth interventions and their underlying factors [22]. Several studies have used UTAUT in the context of eHealth interventions [23,24,25,26,27,28]. The UTAUT model is composed of four key predictors: performance expectancy (PE), effort expectancy (EE), social influence (SI), and facilitating conditions (FC) [29]. Acceptance itself is captured as behavioral intention (BI) to use technology and is predicted by PE, EE, and SI. PE represents the extent to which a person believes they will benefit from using the technology. EE describes the degree of ease to use. SI is defined as the extent to which a person believes that relatives or friends would approve of the use of the technology.

### Objectives

Given the effectiveness of mHealth and CR in patients with CD on the one hand and the lack of utilization and implementation on the other, this study focused on examining the acceptance of mHealth CR and its underlying predictors among patients with CD by using the validated UTAUT model.

The following research questions will be addressed:

1. Which level of acceptance of mHealth CR can be observed among patients with CD?
2. Do individuals with CD differ in acceptance depending on sociodemographic and medical data?
3. Is the proposed extended UTAUT model suitable for assessing the acceptance of digital CR and what are the influencing factors of acceptance in patients with CD?

## 2. Materials and Methods

### 2.1. Participants and procedure

A cross-sectional study was conducted to measure the acceptance of mHealth CR among patients with CD and to assess the influencing factors. Participants in this study were recruited from November 2021 to September 2022 at the Department of Cardiology and Vascular Medicine, West-German Heart, and Vascular Center Essen, University Hospital Essen, in doctors’ offices, self-help groups, and via social media platforms.

Patients with a diagnosis of IHD or CHF were eligible to participate in the study. Additional inclusion criteria were an adult age (≥18 years), sufficient knowledge of the German language, and internet access. Before the start of the survey, all participants provided an electronic declaration of consent. No further exclusion criteria were applied.

The survey was anonymous and voluntary. Participants received no financial compensation. The study was conducted in accordance with the Declaration of Helsinki. The Ethics Committee of the Medical Faculty Essen of the University Duisburg-Essen (19-89-47-BO) approved the study conduct.

On average, answering the survey took *M* = 17 (*SD* = 11) minutes. Of *N* = 527 participants, 60.53% (*N* = 319) completed the survey. *N* = 29 participants were excluded from data analysis because they did not fulfil the inclusion criteria. The excluded patients had no diagnosis of IHD or CDF, but had a diagnosis of peripheral vascular disease. Thus, the final sample included *N* = 290 participants.

### 2.2. Measures

The assessment contained sociodemographic, medical, psychometric, and eHealth-related data. The primary outcome was the acceptance of mHealth CR, conceptualized according to the UTAUT theory [22].

#### 2.2.1. Sociographic and Medical Data

Sociographic data included age, gender, marital status, education, occupational status, ability to work, and place of residence (community size). The medical history contained the following: it was assessed whether the participants had prior myocardial infarction, if they were diagnosed with heart failure, and if they had received prior stent or bypass surgery. Additionally, it was assessed how many flights of stairs patients could climb without shortness of breath or chest pain, which distance they could walk without shortness of breath or chest pain, if they suffered from angina, how often they had to urinate at night, if they had an edema, whether the patients took medication for heart diseases such acetylsalicylacid or cholesterol-lowering drugs, and whether they smoked. In addition, the participants were asked if they had been diagnosed with a mental illness.

#### 2.2.2. Psychometric Data

The Patient Health Questionnaire-8 (PHQ-8) was used to assess depressive symptoms. The PHQ-8 consists of eight items and answers are given on a four-point Likert scale (0 = never to 3 = almost every day). Sum scores ≥ 10 represent severe depressive symptoms [30]. In this study, internal consistency was high (Cronbach’s α = 0.88).

#### 2.2.3. eHealth-Related Data

Internet anxiety was measured with three self-generated items and responses were given on a five-point Likert scale (e.g., “I have concerns about using the internet”, 1 = does not apply to me, 5 = does apply to me). For this scale, internal consistency was acceptable (Cronbach’s α = 0.78).

Participants rated three items regarding their digital confidence (use of digital media, online platforms, and digital devices) on a five-point Likert scale (1 = not confident at all, 5 = very confident). Internal consistency was excellent (Cronbach’s α = 0.94).

Furthermore, participants were asked if they had prior experiences with eHealth interventions. All of the scales used were well-established by their use in previous studies [6,31,32].

#### 2.2.4. Acceptance and UTAUT Predictors

The modified UTAUT questionnaire consists of 13 items, which are scored on a five-point Likert scale (0 = strongly disagree to 4 = strongly agree). The three UTAUT predictors of PE (sample item: “Such an app could help me improve my mental health”), EE (sample item: “Using such an app would not be an additional burden for me”), and SI (sample item: “My cardiology specialist would approve the use of such an app”) were measured with three items each. Acceptance, operationalized as BI, was measured with four additional items. Acceptance (BI) represented the dependent variable in this study. Internal consistency for acceptance (BI) was excellent (Cronbach’s α = 0.90). The three core predictors showed high to excellent internal consistency (Cronbach’s α = 0.91 for PE, α = 0.81 for EE, and α = 0.84 for SI). See Supplementary Material I for the translated version of the modified UTAUT questionnaire used in the study.

### 2.3. Statistical Analysis

Data analysis was performed using SPSS Statistics version 26 (IBM, New York, NY, USA) and the software R (4.0.3). Mean scores for the UTAUT scales (BI, PE, EE, and SI) and sum scores for PHQ-8 were calculated. Moreover, descriptive statistics in the form of mean scores and distributions were performed for other scales and items. In accordance with previous research [23], acceptance (= BI) scores were divided into three categories: low acceptance (scores between 1 and 2.34), moderate acceptance (between 2.35 and 3.67), and high acceptance (between 3.68 and 5). Independent *t*-tests and an ANOVA were used to examine group differences in acceptance (gender, education, prior experiences with mHealth, and mental illness). *p*-values were adjusted for multiple comparisons via Bonferroni correction. Levene’s tests indicated homoscedasticity. Because of the given sample size, normal distribution of residuals was assumed. Multiple hierarchical regression analysis was conducted to determine the predictors of acceptance of mHealth CR. The following predictors were included block-wise: (1) sociodemographic data, (2) medical and psychometric data, (3) eHealth-related data, and (4) UTAUT predictors (PE, EE, and SI). Multicollinearity could not be detected as variance inflation factor (VIF) values were all VIF ≤ 2.6. Visual inspection of qq-plots of the residuals showed no signs of violations against normality, so normal distribution of the residuals could be assumed. Based on a scatter plot of the standardized residuals and the adjusted predicted values, homoscedasticity was verified. The level of significance was set to α < 0.05 for all of the tests, except for the Bonferroni corrected. Effect sizes were reported and interpreted according to Cohen [33], with values around 0.2, 0.5, and 0.8 being considered as small, medium, and large effects, respectively.

## 3. Results

### 3.1. Study Population

In this sample, the mean age was *M* = 57.59 (*SD* = 13.33) years. The youngest participant was 18 years old and the oldest was 94 years old. Here, 39.7% (*N* = 115) of the participants had a prior myocardial infarction, 41.0% (*N* = 119) were diagnosed with CHF, and 52.1% (*N* = 151) had received previous stenting or bypass surgery. Furthermore, 24.8% (*N* = 72) of the individuals were diagnosed with a comorbid mental illness. The most prevalent mental disorders were depression disorders (32, 44.4%), anxiety disorders (17, 23.6%), post-traumatic stress disorder (9, 12.5%), and panic disorder (7, 9.7%). Patients with CD reported low internet anxiety (*M* = 1.36; *SD* = 0.60; range 1–5). Digital confidence was high (*M* = 4.16; *SD* = 0.91; range 1–5). Moreover, 56.2% (*N* = 127) of participants reported prior experiences with eHealth interventions. See Table 1 for a full description of the study population.

### 3.2. Acceptance of mHealth Cardiac Rehabilitation

Overall, acceptance of mHealth CR was high (*M* = 4.05, *SD* = 0.93). Here, 78.3% (*N* = 227) of the participants reported high acceptance, 15.9% (*N* = 46) showed moderate acceptance, and only 5.9% (*N* = 17) reported low acceptance.

Individuals with mental illness reported a significantly higher acceptance of mHealth CR than individuals without a diagnosis (*t*(288) = 3.15, *p_adj_* = 0.007, *d* = 0.43). There was no difference in acceptance between male and female participants, different levels of education, or individuals who had prior experiences with eHealth compared to those without (all *p_adj_* > 0.05).

### 3.3. Predictors of Acceptance of mHealth Cardiac Rehabilitation

Multiple hierarchical regression analysis was applied to determine the influencing factors of acceptance. Data from *n* = 12 participants were excluded because predictors were missing.

Sociodemographic data were included in the first step (*R*^2^ = 0.007, *R*^2^*_adj_* = −0.011, *F*(5272) = 0.40, *p* = 0.847). There were no significant predictors based on the included variables.

Psychometric and medical data were included in the second step (*R*^2^ = 0.130, *R*^2^*_adj_* = 0.103, *F*(8269) = 5.0, *p* < 0.001), which significantly increased the explained variance to 13.0% (∆*R*^2^ = 0.123, *F*(3269) = 35.06, *p* < 0.001). Depressive symptoms (β = 0.34, *p* < 0.001) were significant predictors of acceptance.

The third step included eHealth-related data (*R*^2^ = 0.183, *R*^2^*_adj_* = 0.149, *F*(11,266) = 5.41, *p* < 0.001). Explained variance significantly increased to 18.3% (∆*R*^2^ = 0.05, *F*(3266) = 15.29, *p* < 0.001). In this step, no mental illness (β = −0.27, *p* = 0.047) and digital confidence (β = 0.19, *p* = 0.003) were significant predictors of acceptance.

The three UTAUT predictors were included in the final step of hierarchical regression analysis (*R*^2^ = 0.695, *R*^2^*_adj_* = 0.678, *F*(14,236) = 42.73, *p* < 0.001), which significantly increased the explained variance of the model to 69.5% (∆*R*^2^ = 0.512, *F*(3263) = 146.94, *p* < 0.001). *EE* (β = 0.34), *PE* (β = 0.34), and *SI* (β = 0.26) were significant predictors of acceptance (*p* < 0.001). Table 2 shows the final UTAUT model and its predictors.

## 4. Discussion

The present study investigated the acceptance of digital CR among patients with CD and explored the influencing factors. Overall, acceptance of mHealth CR was high. Individuals with mental illness reported higher acceptance than individuals with a diagnosis. In the extended UTAUT model, acceptance was significantly predicted by depressive symptoms and digital confidence. Consistent with previous findings, the UTAUT factors (EE, PE, and SI) were significant predictors of acceptance and explained a high level of variance. Acceptance did not differ between male and female participants, different levels of education, or individuals who had prior experiences with eHealth compared to those without.

Acceptance was higher in this study than in previous studies that assessed acceptance of eHealth interventions in different patient groups [6,23,34,35,36]. While CR plays an important role in the treatment of CDs, it is underutilized [13]. It is important to inform patients sufficiently about their disease and the associated risk factors. The increasingly shorter hospital stays of patients are related to the lack of information about their disease; this makes it even more important for secondary prevention to involve patients in a CR programs after their hospital stay to create greater awareness and prevent the recurrence of disease events [37]. Digital CR, e.g., mHealth interventions, could thus serve as a useful tool to convey conventional CR. More than 40% of CR facilities had to close partially or completely during the COVID-19 pandemic in 2020 [38], which thus made digital offerings even more important. In Germany, another positive factor driving digitization in healthcare is the new approach that medical apps are prescriptible and the costs are covered by statutory health insurance [39]. To ensure that patients with CD use and benefit from digital CR, facilitators and barriers need to be considered during the development and implementation process. The results of this study support the hypothesis that mHealth CR is met with broad approval from affected patients, which should be utilized to implement new digital healthcare offers.

Other studies investigating the acceptance of eHealth interventions in different samples and patient groups have identified several predictors of acceptance, such as age [23,40], gender [34,40], internet anxiety [25], experience with eHealth interventions [35,40], education [40], and a current or previous diagnosis of mental illness [40]. In contrast with other studies, no difference in acceptance between genders was found in this study. A review from 2019 found eight studies in which there was no significant relationship between gender and eHealth use, while five studies revealed such an association in patients with chronic diseases [41]. In three out of five studies, the female gender was associated with higher usage patterns, while in two other studies, a higher use of eHealth services was found among men. These disparate findings might be able to explain the result of the present study.

This study reveals that depressive symptoms are a positive predictor for acceptance. Furthermore, patients affected by mental illness reported higher levels of acceptance. This relationship could also be observed in other studies [6,36,40]. These findings demonstrate that psychologically burdened individuals are especially open to use new, digital ways of health care offers. However, in future digital CR trials, it would be interesting to assess the potential effect of depressive symptoms on the actual uptake/adherence to digital CR. Digital CR has the advantage that it can also be used by individuals with challenges in managing their daily lives, as they are more barrier-free than face-to-face offers. Chronically ill individuals, such as patients with cardiac disease, are heavily burdened by their illness and experience a reduced quality of life [2]. Reducing this burden should therefore be given a high priority. Moreover, comorbid mental illnesses should be taken into account during the development of patient-centered CR interventions.

Digital confidence emerged as another significant predictor of acceptance, which is in accordance with other studies [32,42,43]. While this relationship is evident, it also underlines the fact that individuals with poor digital confidence or lack of experience with digital media face high barriers to the use of innovative mHealth offers. This difference in capabilities must receive significant consideration in the form of usability testing during the development of new digital CR interventions. It would also be conceivable to hold face-to-face training sessions in advance and to offer in-person support during the course of the intervention.

High acceptance is important for digital CR to be implemented by patients, but other determinants also play a major role in the success of CR. For functional improvement, modifiable factors such as BMI and non-modifiable factors such as age and gender play a role [44]. It is therefore more important to adapt the (digital) CR to the patient’s gender and age, as older patients in particular benefit from a CR and women show a lower adherence to CR programs [44]. Digital CR could be used to implement more individual programs to increase the adherence and the success.

The results of this study support the validity of the UTAUT model for determining the acceptance of mHealth CR. Addressing the influencing factors EE, PE, and SI during the development and implementation process is of high relevance for fostering acceptance. The level of explained variance of the extended UTAUT model was high and comparable to the original UTAUT validation study (70%) [29]. While the three core predictors of the UTAUT model are of high importance, this finding also underlines that additional factors need to be considered to understand and maximize acceptance.

## 5. Limitations

When interpreting the results of this study, the following limitations should be noted. Internet access was a requirement to participate in this study and some of the participants were recruited via the internet. Therefore, it is reasonable to assume that the participants were likely to have a higher willingness and greater interest in internet-related topics, thereby selection bias cannot be ruled out. In addition, all data are based on self-reports, which might represent an additional limitation. Furthermore, the generalizability might be reduced due to the fact that the present sample consisted of younger patients, which might influence the level of acceptance regarding mHealth CR. Lastly, the actual use of mHealth CR needs to be further explored. While acceptance is a predictor of actual usage, the so-called ‘intention-behaviour-gap’ describes the failure to translate intentions into actual behaviour [45]. Therefore, uptake of and adherence to specific mHealth CR offers should be assessed in future studies.

## 6. Conclusions

This study examined the acceptance of digital CR and its underlying predictors in patients with CD. Overall acceptance was high. Patients with comorbid mental illness reported higher acceptance. Important predictors of acceptance were UTAUT predictors of performance expectancy, effort expectancy, and social influence, as well as depressive symptoms and digital confidence. Acceptance is predictive of the actual usage of digital health interventions. The findings of the present study should be considered when implementing digital CR approaches into clinical routine.

## Figures and Tables

**Table 1:** Sociodemographic and medical characteristics stratified by acceptance level.

	Total	High Acceptance	Moderate Acceptance	Low Acceptance
	N (%)	N (%)	N (%)	N (%)
Gender				
Male	141 (48.6)	112 (49.3)	22 (47.8)	7 (41.2)
Female	149 (51.4)	115 (50.7)	24 (52.2)	10 (58.8)
Marital status				
Single	34 (11.7)	23 (10.1)	10 (21.7)	1 (5.9)
In a relationship	41 (14.1)	37 (16.3)	3 (6.5)	1 (5.9)
Married	149 (51.4)	110 (48.5)	25 (54.3)	14 (82.4)
Divorced/separated	41 (14.1)	36 (15.9)	5 (10.9)	-
Widowed	23 (7.9)	20 (8.8)	2 (4.3)	1 (5.9)
Other	2 (0.7)	1 (0.4)	1 (2.2)	-
Education				
No to lower secondary education/Other	66 (22.8)	53 (23.3)	8 (17.4)	5 (29.4)
Secondary education	103 (35.5)	79 (34.8)	16 (34.8)	8 (47.1)
Higher education entrance qualification	55 (19.0)	43 (18.9)	11 (23.9)	1 (5.9)
University education	66 (22.8)	52 (22.9)	11 (3.9)	3 (17.6)
Occupational status				
In education	5 (1.7)	4 (1.8)	1 (2.2)	-
Unemployed	17 (5.9)	16 (7.0)	1 (2.2)	-
Sick leave	17 (5.9)	12 (5.3)	4 (8.7)	1 (5.9)
Partially employed	34 (11.7)	27 (11.9)	4 (8.7)	3 (17.6)
Fully employed	88 (30.3)	73 (32.2)	14 (30.4)	1 (5.9)
Retired	99 (34.1)	75 (33.0)	16 (34.8)	8 (47.1)
Other	30 (10.3)	20 (8.8)	6 (13.0)	4 (23.5)
Unable to work: yes	50 (17.2)	39 (17.2)	8 (17.4)	3 (17.6)
Place of residence (population size)				
Large city (>100,000 residents)	188 (64.8)	156 (68.7)	22 (47.8)	10 (58.8)
Medium sized city (>20,000 residents)	53 (18.3)	38 (16.7)	10 (21.7)	5 (29.4)
Small town (> 5000residents)	26 (9.0)	19 (8.4)	6 (13.0)	1 (5.9)
Rural area (<5000 residents)	23 (7.9)	14 (6.2)	8 (17.4)	1 (5.9)
Flights of stairs				
0	7 (2.4)	6 (2.6)	1 (2.2)	-
1	53 (18.3)	38 (16.7)	10 (21.7)	5 (29.4)
2	93 (32.1)	78 (34.4)	10 (21.7)	5 (29.4)
3	51 (17.6)	42 (18.5)	5 (10.9)	4 (23.5)
4	32 (11.0)	24 (10.6)	6 (13.0)	2 (11.8)
No constraints	54 (18.6)	39 (17.2)	14 (30.4)	1 (5.9)
Walking				
0 to 5 min	19 (6.6)	15 (6.6)	4 (8.7)	-
5 to 10 min	45 (15.5)	43 (18.9)	1 (2.2)	1 (5.9)
10 to 20 min	53 (18.3)	44 (19.4)	5 (10.9)	4 (23.5)
20 to 30 min	39 (13.4)	29 (12.8)	6 (13.0)	4 (23.5)
30 to 40 min	29 (10.0)	22 (9.7)	5 (10.9)	2 (11.8)
More than 40 min	25 (8.6)	14 (6.2)	7 (15.2)	4 (23.5)
No constraints	80 (27.6)	60 (26.4)	18 (39.1)	2 (11.8)
Angina: yes	129 (44.5)	101 (44.5)	22 (47.8)	6 (35.2)
Nocturnal urination (per night)				
Not affected	95 (32.8)	79 (34.8)	12 (26.1)	4 (23.5)
1 to 2 times	157 (54.1)	117 (51.5)	30 (65.2)	10 (58.8)
2 to 3 times	26 (9.0)	21 (9.3)	2 (4.3)	3 (17.6)
More than 3 times	12 (4.1)	10 (4.4)	2 (4.3)	-
Peripheral edema				
Not affected	134 (46.2)	99 (43.6)	26 (56.5)	9 (52.9)
Pressure marks from stockings	104 (35.9)	85 (37.4)	14 (30.4)	5 (29.4)
Visible fluid accumulation	27 (9.3)	24 (10.6)	1 (2.2)	2 (11.8)
Use of compression stockings	25 (8.6)	19 (8.4)	5 (10.9)	1 (5.9)
Medication: yes	201 (69.3)	159 (70.0)	32 (69.6)	10 (58.8)
Smoking: yes	45 (15.5)	37 (16.3)	7 (15.2)	1 (5.9)
Depressive symptoms (PHQ-8 ≥ 10)	128 (44.1)	115 (50.7)	10 (21.7)	3 (17.6)
Total	290 (100.0)	227 (100.0)	46 (100.0)	17 (100.0)

Note. PHQ-8 = Patient Health Questionnaire Depression Scale.

**Table 2:** Hierarchical regression model of acceptance.

Predictors	*B*	β	*t*	*R* ^2^	∆*R*^2^	*p*
(Intercept)	0.22	0.07	0.59			0.554
**Step 1: Sociodemographic data**				**0.007**	**0.007**	
Age	0	−0.05	−1.28			0.201
Gender: Female	0.04	0.05	0.61			0.544
Education: University education	0.00	0.00	0.01			0.994
Education: No to lower secondary education/ Other	−0.09	−0.10	−0.88			0.379
Education: Lower secondary education	0.07	0.07	0.76			0.449
**Step 2: Medical and psychometric data**				**0.130**	**0.123**	
Mental illness: No	−0.13	−0.14	−1.63			0.104
Depressive symptoms (PHQ-8)	0.02	0.10	2.28			0.024
Flights of stairs	0.05	0.07	1.80			0.074
**Step 3: eHealth-related data**				**0.183**	**0.050**	
Digital confidence	−0.00	−0.00	−0.00			0.997
Internet anxiety	0.11	0.07	1.74			0.083
Prior experiences with mHealth interventions: No	0.02	0.02	0.32			0.747
**Step 4: UTAUT predictors**				**0.695**	**0.512**	
Effort expectancy	0.37	0.34	6.85			<0.001
Performance expectancy	0.33	0.34	6.27			<0.001
Social influence	0.26	0.25	4.93			<0.001

Note. *N* = 278. In Steps 2, 3, and 4, only the newly included variables are presented. *B* = unstandardized beta. β = standardized beta. *t* = Test statistic. *R*² = determination coefficient. ∆*R*^2^ = changes in *R*^2^. PHQ-8 = Patient Health Questionnaire Depression Scale.

## Data Availability

The data supporting the presented results are available upon reasonable request to the corresponding author.

## Supplementary Materials

The following supporting information can be downloaded at: https://www.mdpi.com/article/10.3390/jcdd10040174/s1. Modified UTAUT questionnaire—English Version.
