# Supplementary material for: Determining the Acceptance of Digital Cardiac Rehabilitation and Its Influencing Factors among Patients Affected by Cardiac Diseases
Source: J Cardiovasc Dev Dis. 2023 Apr 17;10(4):174. doi: 10.3390/jcdd10040174 (PMC10144862; doi:10.3390/jcdd10040174)
Supplement: Supplementary file 1 [file jcdd-10-00174-s001.zip › jcdd-2279010-supplementary.pdf]

Supplementary Material I - Determining the Acceptance of Digital Cardiac Rehabilitation and its Influencing Factors among Patients affected by Cardiac Diseases

Modified UTAUT questionnaire – English Version

Items of the modified UTAUT questionnaire

1. I would like to try such an app
2. I would use such an app if it was offered to me
3. I would use such an app if my health insurance covered the costs
4. I would recommend such an app to acquaintances of mine with heart problems
5. People close to me would approve the use of such an app
6. My family doctor would approve the use of such an app
7. My cardiology specialist would approve the use of such an app
8. Such an app could improve my general well-being
9. Such an app could help me with stress and stress management
10. Such an app could help me improve my mental health
11. Using such an app would not be an additional burden for me
12. Such an app would be easy for me to use and understand
13. I could incorporate the use of such an app into my daily life

Note: Translated items only for publication purpose. The original items were presented in German.
